# The Lantibiotic Lacticin 3147 Prevents Systemic Spread of *Staphylococcus aureus* in a Murine Infection Model

**DOI:** 10.1155/2012/806230

**Published:** 2012-01-12

**Authors:** Clare Piper, Pat G. Casey, Colin Hill, Paul D. Cotter, R. Paul Ross

**Affiliations:** ^1^Department of Microbiology, University College Cork, College Road, Cork, Ireland; ^2^Alimentary Pharmabiotic Centre, Cork, Ireland; ^3^Food Biosciences Department, Teagasc Food Research Centre, Moorepark, Fermoy, Cork, Ireland

## Abstract

The objective of this study was to investigate the *in vivo* activity of the lantibiotic lacticin 3147 against the luminescent *Staphylococcus aureus* strain Xen 29 using a murine model. Female BALB/c mice (7 weeks old, 17 g) were divided into groups (*n* = 5) and infected with the Xen 29 strain via the intraperitoneal route at a dose of 1 × 10^6^ cfu/animal. After 1.5 hr, the animals were treated subcutaneously with doses of phosphate-buffered saline (PBS; negative control) or lacticin 3147. Luminescent imaging was carried 3 and 5 hours postinfection. Mice were then sacrificed, and the levels of *S. aureus* Xen 29 in the liver, spleen, and kidneys were quantified. Notably, photoluminescence and culture-based analysis both revealed that lacticin 3147 successfully controlled the systemic spread of *S. aureus* in mice thus indicating that lacticin 3147 has potential as a chemotherapeutic agent for *in vivo* applications.

## 1. Introduction

*Staphylococcus aureus* is one of the most significant bacterial pathogens and can cause diseases ranging from minor and surgical site infections [1] to potentially life-threatening endocarditis [2–4] and bacteraemia [5–8]. It is a particular problem in hospitals as a consequence of the emergence and dissemination of multidrug-resistant forms such as methicillin-resistant *S. aureus* (MRSA), vancomycin intermediate susceptibility *S. aureus* (VISA), and heterogenous VISA (hVISA). The prevalence of these antibiotic resistant forms means that the discovery of novel chemotherapeutic agents to combat these pathogens is of key importance [9, 10]. The lantibiotics (lanthionine-containing antibiotics [11]) are a group of posttranslationally modified antimicrobial peptides of which nisin and lacticin 3147 are among the most extensively investigated. A number of lantibiotics have been noted to exhibit potent antimicrobial activity against staphylococci of clinical relevance. In agar diffusion assays, the type I lantibiotics epidermin, Pep5, epicidin K7, and epilancin 280 display impressive levels of activity against coagulase negative staphylococci (CNS) [12], and it has been suggested that their potential could be exploited to prevent the colonization of medical devices [12]. Nisin has also been shown on several occasions to possess significant anti-*Staphylococcus* activity. When tested against 20 MRSA strains, one study revealed that the minimum inhibitory concentration (MIC) of nisin A ranged between 1.5 and 16 mg/L [13], while a more recent investigation revealed MICs of 0.5–4.1 mg/L [14]. The *in vitro* activity of other forms of nisin (nisin F, Q, and Z) against MRSA has also recently been highlighted [15]. The *in vivo* activity of a number of lantibiotics against staphylococci has also been investigated. The effectiveness of the epidermin-like mutacin B-Ny266 was tested on mice infected by intraperitoneal (IP) injection with 3.1 × 10^7^ cfu of *S. aureus* Smith/mouse. Immediately after injection, mutacin B-Ny266 was administered, also via the IP route, at concentrations of 1–10 mg/kg of mouse and was found to be protective [16]. More recently, it has been established that microbisporicin, in addition to having potent* in vitro *activity (MIC ≤ 0.13 *μ*g/mL), effectively controls murine septicemia caused by *S. aureus* in female CD-1 mice (23–25 g). The mice were infected via the IP route with 1 × 10^6^ cfu of *S. aureus* Smith 819 ATCC 19636 in 0.5 mls gastric hog mucin. Microbisporicin was then administered intravenously or subcutaneously (SC) 10–15 mins after infection at final concentrations of 10–15 mg/L [17]. The effective dose 50 (ED_50_) of microbisporicin was found to be 2.1 mg/kg regardless of whether it was administered via IV or SC. ED_50_ values were determined on the bases of survival of the mice to the seventh day. Higher doses of microbisporicin (≥200 mg/kg) led to the survival of all animals treated and were nontoxic [16]. Nisin F effectively controlled the MRSA strain, *S. aureus* K, in immunocompromised Wistar rats following the introduction of 4 × 10^5^ *S. aureus* cells into the nostrils of the rats for 4 consecutive days before treating with 8192 arbitrary units (AU) of nisin F intranasally for the subsequent 4 days [18]. In contrast, however, when 1 x10^8^ *S. aureus* Xen 36 cells were injected intraperitoneally, the administration of a lower concentration of nisin F (640 AU) after 4 hours succeeded in inhibiting the growth of the pathogen for only 15 minutes after which time the pathogen reemerged [19]. Finally, short- and long-term *in vivo* studies with mersacidin established that this lantibiotic quite effectively inhibited MRSA introduced intranasally into immunocompromised (hydrocortisone-treated) BALB/C mice [20]. For the short term trial, the mice were infected on days 5, 7, and 9 with 3 × 10^2^–10^4^ cfu of the *S. aureus* strain. The mice were then treated intranasally with mersacidin (1.66 mg/kg per treatment) twice a day on days 10, 11, and 12. For the longer trial, the mice were challenged with *S. aureus* on days 5, 7, 9, 30, 32, and 34 and subsequently treated with mersacidin on days 35, 36, and 37. In both cases the mersacidin treatment successfully inhibited MRSA-induced rhinitis [20]. Notably, a comparison of the *in vitro* and *in vivo* activity of mersacidin against a number of MRSA strains indicates that mersacidin more effectively inhibits *S. aureus in vivo *[21].

Lacticin 3147 is the most extensively investigated of the two peptide lantibiotics. These peptides are active as a consequence of the synergistic activity of two lanthionine-containing peptides [22, 23]. Lacticin 3147 has been found to exhibit potent *in vitro* activity against a range of pathogenic bacteria including *Clostridium difficile*, vancomycin-resistant enterococci, *Propionibacterium acne, *penicillin-resistant* Pneumococcus,* and *Streptococcus mutans *[14, 24–26] as well as pathogenic mycobacteria such as *Mycobacterium avium* subsp *paratuberculosis *and *Mycobacterium tuberculosis* H37Ra [27]. Of greatest relevance to this study is the fact that lacticin 3147 possesses anti-*Staphylococcus* activity. The lantibiotic itself, when incorporated into a teat seal, protects against *S. aureus*-associated bovine mastitis [28, 29], while use of a lacticin 3147-producing *Lactococcus lactis* DPC 3251 within a teat dip inhibits *S. aureus* both *in vitro* and also *in vivo *[30]. The *in vitro* activity of lacticin 3147 against clinical MRSA isolates has also been established with MICs ranging from 1.9 to 15.4 mg/L [14].

Despite lacticin 3147 being one of the most extensively studied lantibiotics, its ability to control a systemic infection caused by *S. aureus*, or indeed any other pathogen, has not been investigated. Here we address this issue using BALB/c mice infected via the IP route with *S. aureus* Xen 29, a strain of methicillin sensitive *S. aureus* (MSSA) that has been genetically modified to express the *Photorhabdus luminescens lux *genes to facilitate *in vivo* imaging. The ability of subcutaneously administered lacticin 3147 to control infection was assessed by *in vivo* imaging and microbiological analysis of the organs of sacrificed animals.

## 2. Materials and Methods

### 2.1. Antimicrobial Activity Assays

The *in vitro* activity of lacticin 3147 and vancomycin (employed as a positive control) against *S. aureus* Xen 29 was assessed through MIC determination assays carried out in triplicate as described previously [14] with purified lacticin 3147, prepared via HPLC, again as described previously [14]. Vancomycin was obtained from Sigma Aldrich.

### 2.2. Inoculum Preparation

*S. aureus* Xen 29 (derived from the parental pleural isolate *S. aureus* 12600; Xenogen Corporation, Almeda, CA) possesses a copy of the modified *luxABCDE* operon of *P. luminescens *integrated at a single site on the chromosome. *S. aureus* Xen 29 was cultured overnight in brain heart infusion (BHI) broth aerobically at 37°C from an isolated colony growing on BHI agar containing 200 *μ*g/mL kanamycin. On the day of the trial, the overnight culture was subcultured (1 : 100 dilution) into fresh BHI and grown to log phase (OD_600nm_ of 0.5). This culture was diluted to facilitate the ultimate administration of the culture in the form of a 1 × 10^6^ cfu/100 *μ*L dose in 0.5% hog gastric mucin (Sigma Aldrich).

### 2.3. Mouse Peritonitis Model

Mice were fed a standard rodent diet *ad libitum* and all animal studies were approved by the Animal Experimentation Ethics Committee. 13 BALB/c female mice (7 weeks old, 15 g ± 2 g in weight) were divided into 3 groups (A, B, C; *n* = 3, 5, and 5, resp.). At T_0_ mice in groups A–C received the 1 × 10^6^ cfu dose (100 *μ*L volume) via the IP route in 0.5% gastic hog mucin (Sigma Aldrich). At T_1.5 hrs_, the mice in group C were administered lacticin 3147 (50.85 mg/kg of Ltn*α* and 43.8 mg/kg of Ltn*β*, corresponding to 30.76 mM lacticin 3147/kg) in a single dose and a second dose at T_3hrs_ (25.425 mg/kg Ltn*α* and 21.90 mg/kg Ltn*β*; 15.382 mM lacticin 3147/kg). Vancomycin (50 mg/kg; 33.6 mM/kg) was administered at T_1.5 hrs_ and at T_3hrs_ to the mice in group B while the mice in group A received PBS (once) as a control. Both antimicrobials and PBS were adminisitered subcutaneously in 100 *μ*L doses. *In vivo *imaging was carried out at two time points that is, 3 hours and 5 hours postinfection. Mice were anaesthetized for bioluminescent imaging *via* the inhalation of aerosolized isoflurane mixed with oxygen. The mice were then transferred to the IVIS chamber ventral side up, and luminescence was measured over a 3-to-5 mins exposure time. The imaging system measures the number of photons reaching each detector of the charged-couple device camera, and the IVIS software translates these data into false color images that display regions of intense luminescence with red, moderate luminescence in yellow and green and mild luminescence in blue. The images contained herein are photographic images with an overlay of bioluminescence that uses this computer-generated color scale [31]. The mice were euthanized approximately 6 hours postinfection. Liver, kidneys, and spleen were extracted. These organs were mechanically disrupted and serial dilutions made which were subsequently plated in 100 *μ*L volumes on TSA − Kan^200*μ*g/mL^ plates in order to enumerate the staphylococci present in each organ.

### 2.4. Quantification of Luminescence

Luminescent images were quantified with IVIS imaging software. The total flux (number of photons/s/cm^2^) was calculated by a user defined area (region of interest) covering the infection site. The flux was averaged across all mice from each respective group. The reduction in luminescence was quantified and represents a comparison with the luminescence from mice administered phosphate-buffered saline control at the same time point.

### 2.5. Statistical Analysis

The mean and standard error of the mean (SEM) of the luminescence at the final time point and bacterial counts for the mice were calculated for all groups. Differences in the bioluminescence and bacterial counts analyzed through a one-way analysis of variance, followed by the Holm-Sidak posttest (Sigma Stat, version 3.5).

## 3. Results/Discussion

### 3.1. Assessment of the *In Vivo* Activity of Lacticin 3147 against *S. aureus* Xen 29 Using a Murine Peritonitis Model

**Figure 1:**
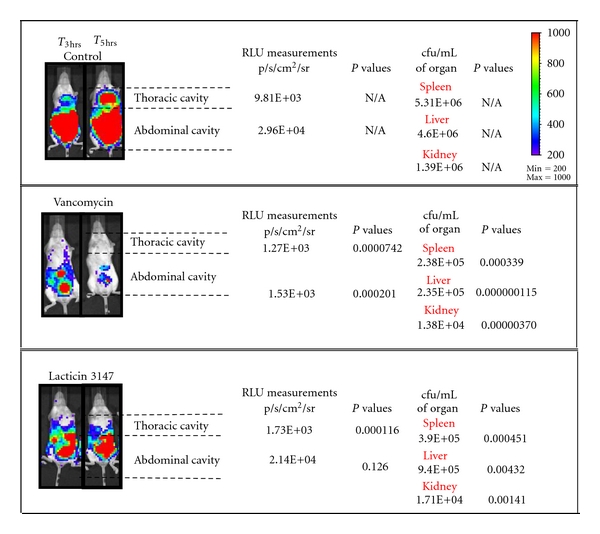
Impact of lacticin 3147 on the systemic spread of *S. aureus* Xen 29 in mice. Images are of representative mice from each group 3 and 5 hours postinfection. Values (i.e., RLU measurements and cfu/mL of organs) represent averages of data collected at T_5hrs_, and *P* values refer to the significance of differences between the treated and untreated equivalents at T_5hrs_ as determined by one-way analysis of variance, followed by the Holm-Sidak posttest. The imaging system depicts false-color images representative of different levels of total flux. False color imaging represents intense luminescence in red, moderate luminescence in green, and low level luminescence in blue/purple.

The ability of subcutaneously injected lacticin 3147 to control a systemic *S. aureus* infection following the introduction of the pathogen into the murine peritoneal cavity was investigated. This involved *in vivo* imaging to detect levels of light emitted by the pathogen within mice and through the postmortem microbiological analysis of organs. Negative and positive controls were employed in the form of mice treated with PBS and the glycopeptide antibiotic vancomycin, respectively. The target strain* S. aureus* Xen 29 is a methicillin sensitive isolate which has been employed previously to facilitate an investigation of acute *in vivo* infections [32–36]. Prior to commencement of the study, the *in vitro* sensitivity of Xen 29 to lacticin 3147 was assessed. The corresponding MIC values were 1.013 mg/L and 19.1 mg/L for vancomycin and lacticin 3147, respectively (Table 1). For *in vivo* studies, mice received a dose of 1 × 10^6^ cfu and, 1.5 hrs postinfection, were administered lacticin 3147 (50.85 mg/kg of Ltn*α* and 43.8 mg/kg of Ltn*β*), vancomycin (50 mg/kg), or PBS. At T_3hrs_, the mice were subject to IVIS imaging, and second doses of lacticin 3147 (25.425 mg/kg and 21.90 mg/kg) and vancomycin (50 mg/kg) were administered to the relevant mice. IVIS analysis of the progression of the *S. aureus* Xen 29 infection showed that the pathogen spreads systemically and eventually also occupies the thoracic cavity in mice injected with PBS 5 hrs (T_5hrs_) after injection of the pathogen. A significant (*P* = 0.000116) reduction in the RLU measurements corresponding to the thoracic region of the lacticin 3147 treated group was evident when compared to that of the PBS (negative) control group (Figure 1) at this time point highlighting the ability of the lantibiotic to prevent systemic spread of the *S. aureus* Xen 29 infection. In contrast, lacticin 3147 does not significantly reduce RLU values corresponding to the peritoneal cavity relative to the control. It may be that lacticin 3147 is deficient in penetrating the peritoneal cavity (Figure 1). To further ascertain lacticin 3147 efficacy, culture-based analysis of staphylococcal levels in the organs was determined after the mice were sacrificed. This analysis further highlighted the success of lacticin 3147 in controlling systemic infection. Lacticin 3147 treatment resulted in a significant reduction (*P* < 0.05 in all cases; Figure 1) in pathogen numbers in the liver, spleen, and kidneys of the mice treated relative to the PBS-treated controls (Figure 1).

As expected, vancomycin brought about a significant reduction in *S. aureus* levels relative to the PBS-treated controls as determined by both bioimaging and culture-based analysis. Notably, numbers of *S. aureus* in the spleens of lacticin 3147- and vancomycin-treated mice were statistically indifferent. However, vancomycin treatment more successfully lowered *S. aureus* numbers in the liver and kidneys. While both lacticin 3147 and vancomycin bind lipid II, [10, 37, 38] differences exist with respect to their mechanism of action. Vancomycin binds to the C-terminal D-Ala-D-Ala motif of the pentapeptide of lipid II [10] whereas, on the basis of similarities between Ltn*α* and mersacidin, it is proposed that lacticin 3147 binds to the sugar phosphate head group of lipid II [39]. Furthermore, lacticin 3147 is also capable of forming pores in the membranes of target cells [37, 38]. It should be noted that while similar mg/kg doses of lacticin 3147 and vancomycin were employed in this study, our *in vitro* investigations established that vancomycin is 19 times more potent than lacticin 3147 against Xen29 (MIC values; 1.013 mg/L and 19.1 mg/L of vancomycin and lacticin 3147, resp.). Thus the dose of vancomycin administered *in vivo* corresponded to 100-fold that of the *in vitro* MIC whereas lacticin 3147 was administered at a level 8-fold greater than its *in vitro* MIC. This may explain the enhanced ability of vancomycin with respect to clearance of Xen 29 from the peritoneal cavity. This is the first occasion upon which the impact of lacticin 3147 against a systemic infection has been assessed and thus it is also the first instance of its administration subcutaneously. It may be that lacticin 3147 cannot travel to the peritoneum to eradicate the infection but can prevent the spread of infection throughout the blood stream. As stated previously, mersacidin has successfully been shown to inhibit a systemic MRSA infection in mice when administered via the subcutaneous route [20]. However, mersacidin is a one-component lantibiotic and is also globular which may provide facile delivery through the skin. It, like vancomycin, is quite a small peptide with a molecular weight of 1, 825 Da [40]. Lacticin 3147 consists of 2 peptides with molecular weights of 3305 Da (Ltn*α*) and 2847 Da (Ltn*β*), and it may be that the larger size of the individual peptides or a specific difficulty relating the transport of one of the components to the peritoneal cavity may be an issue. Mutacin B-Ny266 has also been shown to protect against *S. aureus* in the peritoneum, but this lantibiotic was administered intraperitoneally, and thus transfer to the site of infection was not an issue [16].

### 3.2. Conclusion

In conclusion, here we have provided evidence that lacticin 3147 could be employed to treat systemic infections. Both culture- and bioluminescence-based analyses reveal that the lantibiotic significantly reduces numbers of the *S. aureus* Xen29 relative to the negative control by preventing the dissemination of the pathogen. Although these results are more promising than those described when nisin F was employed in a similar manner (19), differences with respect to the strains of *S. aureus* employed, concentrations of lantibiotic, and other factors mean that a direct comparison of outcomes is not possible. While further investigations are required, over longer periods of time, to more extensively assess the clinical potential of lacticin 3147, it is worth noting that lacticin 3147 possesses many physicochemical properties that favour its *in vivo *application. These include excellent activity over a broad pH range, especially at physiological pH (pH 7), the absence of cytotoxicity towards eukaryotic cells [41], its broad spectrum of activity at nanomolar concentrations [42], its alternative mode of action [38], and the presence of (methyl)lanthionine bridges that confer structural rigidity to lantibiotics and reduce proteolytic attack [43]. These properties, accompanied by its ability to inhibit a systemic *S. aureus* infection, make lacticin 3147 a promising candidate for potential applications in human medicine.

## Figures and Tables

**Table 1:** The standard deviation in all cases is 0 reflecting identical triplicate results.

*S. aureus* Xen 29	MIC (mg/L)
Vancomycin	1.013
Lacticin 3147	19.1
